# The first complete mitochondrial genome of Matsucoccidae (Hemiptera, Coccoidea) and implications for its phylogenetic position

**DOI:** 10.3897/BDJ.10.e94915

**Published:** 2022-11-09

**Authors:** Kai Hu, Sanpeng Yu, Niannian Zhang, Maojuan Tian, Qiming Ban, Zhongliang Fan, Jiansheng Qiu

**Affiliations:** 1 Guizhou Academy of Forestry, Guiyang, China Guizhou Academy of Forestry Guiyang China; 2 Forestry Administration of Wangmo County, Qianxinan, China Forestry Administration of Wangmo County Qianxinan China; 3 Forestry Administration of Huichuan District, Zunyi, China Forestry Administration of Huichuan District Zunyi China

**Keywords:** mitogenome, scale insect, phylogenetic analysis, secondary structure, Sternorrhyncha

## Abstract

The mitochondrial genome (mitogenome) has been extensively used to better understand the phylogenetic relationships within the hemipteran suborder Sternorrhyncha, but sequenced mitogenomes remain unavailable for the entire family Matsucoccidae to date. To address this, here we sequenced the complete mitogenome of *Matsucoccusmatsumurae*; the first for this family. The mitogenome is 15,360 bp in size and comprises the typical set of 37 mitochondrial genes and a large non-coding region (AT-rich region). Gene order, nucleotide composition and codon usage of protein-coding genes (PCGs) of *M.matsumurae* differ considerably from those of the other two sequenced Coccidae species. All PCGs were initiated by the ATN start codons and ended with the TAA/G or single T-- stop codons. Nine transfer RNA genes could be folded into typical clover-leaf secondary structures. The length and AT content of the ribosomal RNA genes are highly conserved in the Coccoidea mitogenomes. In contrast, the AT-rich control region is highly variable in size and in the number of tandem repeats. The sliding window analysis showed that the *cox1* gene is the most conserved amongst the 13 PCGs, while the ratios of non-synonymous to synonymous substitution rates indicated that the evolution of this mitogenome has been dominated by positive selection. Phylogenetic analyses, based on nucleotide sequence data of 37 mitochondrial genes and amino acid sequence data of 13 PCGs using Bayesian Inference and Maximum Likelihood methods, showed that Matsucoccidae diverged before the Coccidae.

## Introduction

The family Matsucoccidae was erected in 1984 by Koteja (Koteja 1984, Koteja 1990) within the superfamily Coccoidea. It belongs to the Archaeococcoidae clade of the infraorder Coccomorpha, suborder Sternorrhyncha, order Hemiptera. So far, a total of 38 species in two genera of Matsucoccidae have been recorded worldwide (García Morales et al. 2018). Except for six fossil species described from Baltic amber, all 32 extant species exclusively occur on *Pinus* spp. in the Holarctic and Neotropical Regions (García Morales et al. 2018). Amongst them, some species are amongst the most destructive pests of pine trees, causing significant economic losses (McKenzie et al. 1948, Furniss and Carolin 1977, Foldi 2004, Lim et al. 2013, Liu et al. 2014). Based on the fossil evidence and morphological characteristics, Matsucoccidae is considered to be one of the most primitive families amongst the archaeococcoids and may even represent the most ancient of all scale insects (Coccoidea) (Beardsley 1968, Foldi 2004, Koteja and Azar 2008, Hodgson and Hardy 2013, Wang et al. 2016). However, this lacks the support of molecular data.

In insects, the mitochondrial genome (mitogenome) is typically a covalently closed circular double-stranded DNA molecule, usually 15 ~ 18 kb in length, that encodes 37 genes, including 13 protein-coding genes (PCG), two ribosomal RNA genes (rRNA) and 22 transfer RNA genes (tRNA) (Boore 1999, Cameron 2014). In addition, the mitogenome usually includes a non-coding region of variable length that plays a regulatory role in the transcription and replication, namely, the mitochondrial control region (AT-rich region) (Clayton 1982, Boore 1999). The mitogenome, in whole or part, has been widely used as a molecular marker to study population genetics, phylogeny and genetic evolution of insects (Hu et al. 2019).

Currently (23 April 2022), there are no available mitogenomes for Matsucoccidae. In this study, we sequenced, annotated and analysed the mitogenome of *Matsucoccusmatsumurae* (the type species of Matsucoccidae) in detail. Furthermore, we sampled all sternorrhynchan superfamilies to investigate the phylogenetic position of Matsucoccidae and also provide insight into the superfamily-level phylogenetic relationships within the suborder Sternorrhyncha.

## Materials and methods

### Sample collection and DNA extraction

**Figure 1.**
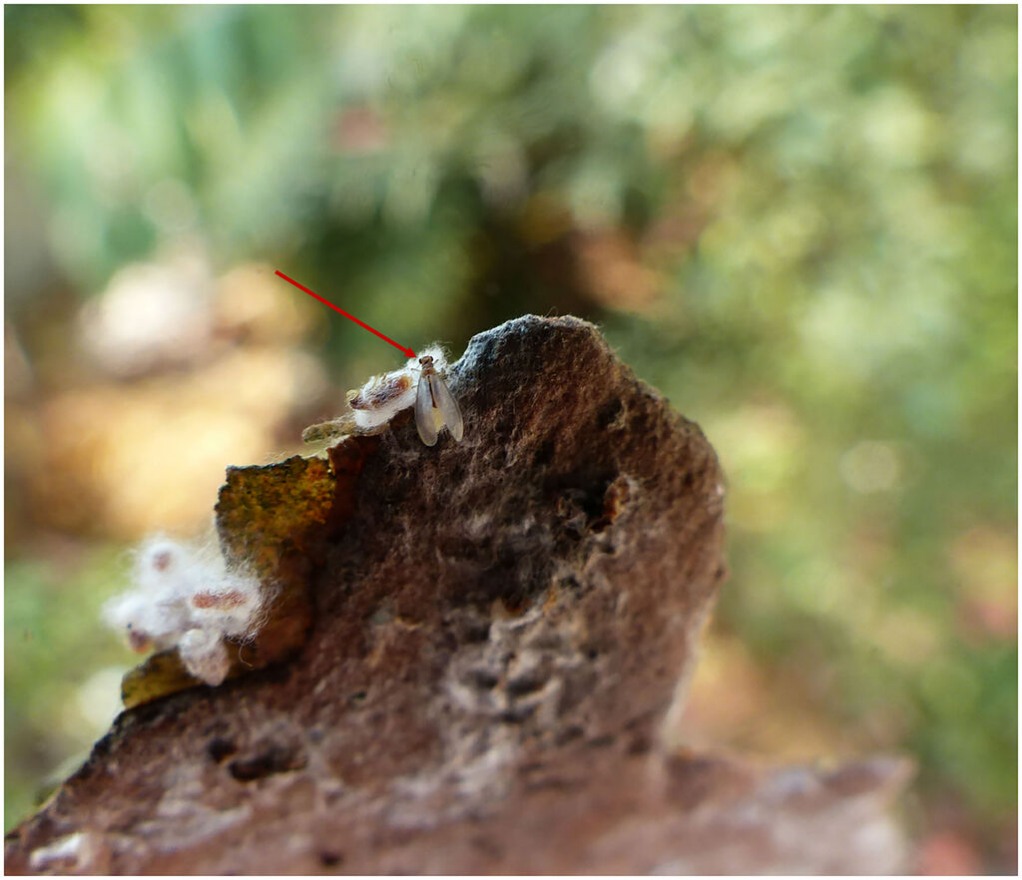
An adult male of *Matsucoccusmatsumurae* on *Pinusmassoniana* (Guizhou Province, China).

Adult specimens of *M.matsumurae* (Fig. 1) were collected from *Pinusmassoniana* in Guizhou Province, China (Suiyang County, 107°2′52.06″E, 27°52′10.02″N, September 2021). All fresh specimens were preserved in 100% ethyl alcohol immediately after collection in the field and deposited at -20°C in the laboratory of Guizhou Academy of Forestry, Guiyang, Guizhou. Identification of the specimens was based on morphological characteristics (Li 2016). The total DNA was extracted from thoracic muscles using the Biospin Insect Genomic DNA Extraction Kit (BioFlux) following the manufacturer’s instructions. Voucher specimens are stored in the entomological collection of the Guizhou Academy of Forestry.

### Mitogenome sequencing and assembly

The whole genomic DNA of *M.matsumurae* was sequenced using next-generation sequencing (Illumina HiSeq X10, Biomarker Technologies Corporation, Beijing, China). About 2.13 Gb clean data were assembled into a complete circular mitogenome using NOVOPlasty v.4.3.1 (Dierckxsens et al. 2017) with the *cox1* gene of *Saissetiacoffeae* (Lu et al. 2020) as the seed sequence.

### Mitogenome annotation and analyses

The annotation of mitogenome was conducted using Geneious Prime v.2022.0.1 (Biomatters, Auckland, New Zealand). The locations and sequences of tRNA genes were determined by the MITOS Web Server (http://mitos2.bioinf.uni-leipzig.de/index.py) (Donath et al. 2019) and comparison with homologous mitogenome sequences. Secondary structures of tRNAs were plotted using Adobe Illustrator CC2017 according to MITOS results and manual predictions (following the expected cloverleaf secondary structure of tRNA genes). Two rRNA genes and 13 PCGs were identified, based on their alignments with the other scale insect (Coccoidea) mitogenome sequences. The mitogenomic circular map was depicted with the help of OrganellarGenomeDRAW (OGDRAW) (https://chlorobox.mpimp-golm.mpg.de/OGDraw.html) (Greiner et al. 2019).

The organisation tables, nucleotide composition and relative synonymous codon usage (RSCU) of the mitogenomes of Coccoidea species were calculated and produced using PhyloSuite v.1.2.2 (Zhang et al. 2020). The nucleotide diversity (Pi) analyses of 13 PCGs and two rRNA genes of the Coccoidea species and a sliding window analysis (a sliding window of 200 bp and step size of 20 bp), were conducted by DnaSP v.6.0 (Rozas et al. 2017). The non-synonymous substitution rates (Ka), synonymous substitution rates (Ks) and ratios of non-synonymous to synonymous substitution (Ka/Ks) for each of the concatenated 13 PCGs of the Coccoidea mitogenomes were also calculated by DnaSP v.6.0. Tandem repeats of the control region were identified with the Tandem Repeats Finder server (https://tandem.bu.edu/trf/trf.html) (Benson 1999).

### Phylogenetic analysis

A total of 34 mitogenomes from five superfamilies of Hemiptera were used for the phylogenetic analyses (Table 1). Of these, 32 species belong to all four superfamilies of Sternorrhyncha (the ingroup), while the remaining two species from the superfamily Fulgoroidea were chosen as outgroups. Nucleotide sequences (without stop codons) for the 13 PCGs were aligned using MAFFT v.7 (Katoch and Standley 2013) with the L-INS-i (accurate) strategy and codon alignment mode (Code table: Invertebrate mitochondrial genetic codon), rRNA and tRNA gene sequences were aligned using MAFFT v.7 (Katoch and Standley 2013) with the G-INS-I algorithm (which takes account of the secondary structure of rRNA and tRNA genes) and normal alignment mode and amino acid sequences of 13 PCGs were aligned using the -auto strategy and normal alignment mode. Ambiguously aligned areas were removed using Gblocks v.0.91b (Talavera and Castresana 2007), respectively. Individual gene alignments were concatenated using PhyloSuite v.1.2.2 (Zhang et al. 2020). For amino acid sequence data, Bayesian Inference (BI) phylogenetic analysis were conducted using PHYLOBAYES MPI v.1.5c (Lartillot et al. 2013) in the CIPRES Science Gateway (Miller et al. 2010) which employs the site-heterogeneous model CAT + GTR. Two independent Markov Chain Monte Carlo (MCMC) chains were run and the analysis was stopped when the two runs had satisfactorily converged (maxdiff. fell below 0.3). A consensus tree was computed from the remaining trees combined from two runs after the initial 25% trees from each MCMC chain run were discarded as burn-in. For nucleotide sequence data, the best partitioning scheme and nucleotide substitution models for Maximum Likelihood (ML) and BI phylogenetic analyses were selected with PartitionFinder v.2 (Lanfear et al. 2017) using the Bayesian Information Criterion (BIC) (Suppl. materials 1, 2). ML analyses were conducted using IQ-TREE v.1.6.3 (Nguyen et al. 2015) under the ultrafast bootstrap (UFB) approximation approach (Minh et al. 2013) with 5,000 replicates. BI analysis was performed using MrBayes v.3.2.7a (Ronquist et al. 2012) in the CIPRES Science Gateway server (Miller et al. 2010) with four chains (one cold chain and three hot chains). Two independent runs of 5,000,000 generations were carried out with sampling every 1,000 generations. The first 25% of trees were discarded as burn-in. After the average standard deviation of split frequencies fell below 0.01, stationarity was assumed.

## Results and discussion

### Mitogenome organisation and nucleotide composition

**Figure 2.**
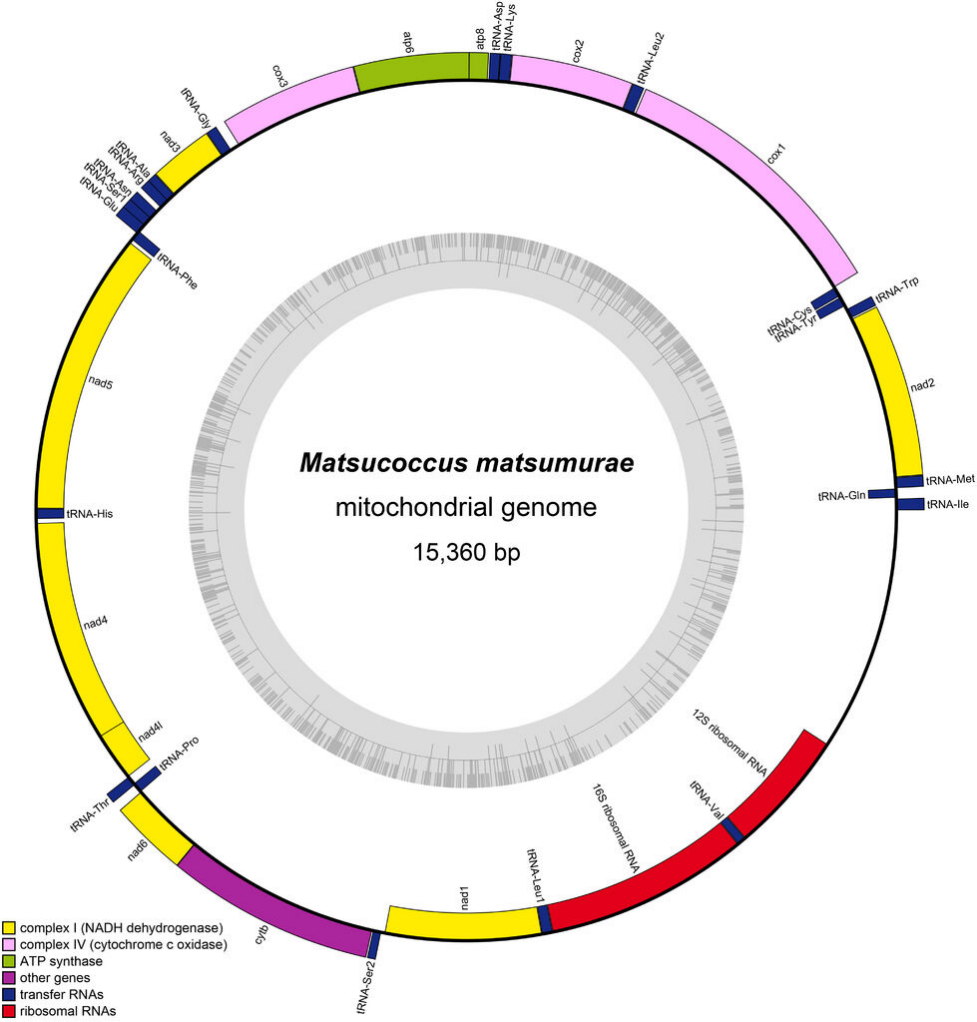
Circular map of the mitogenome of *Matsucoccusmatsumurae*. The outer circle shows the gene map of *M.matsumurae*, with the genes outside the map encoded on the major strand (H-strand), whereas genes on the inside of the map are encoded on the minor strand (L-strand). Genes are represented by different colour blocks.

**Figure 3.**
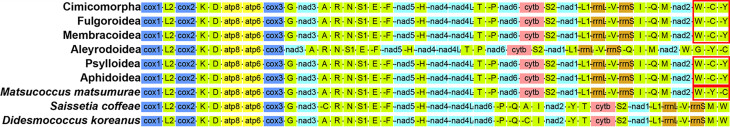
Gene orders in Hemiptera mitogenomes. With the exception of Coccoidea (*Didesmococcuskoreanus*, *Matsucoccusmatsumurae* and *Saissetiacoffeae*), each superfamily (plus the infraorder Cimicomorpha) is represented by one species. Genes shown with “-“ signs are located on the minor strand (L-strand), while others are located on the major strand (H-strand).

The complete mitogenome of *M.matsumurae* is a closed circular double-stranded DNA molecule (Fig. 2, Suppl. material 3), 15,360 bp in length. This is medium-sized amongst the available mitogenomes of Coccoidea: from 15,143 bp for *Didesmococcuskoreanus* to 15,389 bp for *Saissetiacoffeae* (Lu et al. 2020, Xu et al. 2021). The newly-sequenced mitogenome encodes 37 genes (13 PCGs, 22 tRNAs and two rRNAs) and an A+T-rich region (control region), which is a typical architecture for bilaterian animal mitogenomes (Boore 1999). The gene order of *M.matsumurae* (Matsucoccidae) differs considerably from that of the other two Coccidae species (Lu et al. 2020, Xu et al. 2021), but it is comparatively similar to gene orders of most Cimicomorpha, Fulgoroidea, Membracoidea, Psylloidea and Aphidoidea species (only the tRNA cluster of *trnW*–*trnC*–*trnY* was rearranged to *trnW*–*trnY*–*trnC*) (Fig. 3). Amongst the three scale insect species, twenty-three genes (nine PCGs and 14 tRNAs) are encoded on the majority strand (H strand) and the remaining 14 genes (four PCGs, eight tRNAs and two rRNAs) are encoded on the minority strand (L strand) (Fig. 3). A total of 85 overlapping nucleotides were found in nine pairs of neighbouring genes, with the longest identified overlap between the *trnN* and *trnS1* (22 bp). Furthermore, there are 193 intergenic nucleotides dispersed across 16 gene boundaries. The longest intergenic region (103 bp) is located between *cox3* and *trnG*.

The overall nucleotide composition of the *M.matsumurae* mitogenome is 47.2% A, 6.4% C, 0.6% G and 45.8% T. It therefore exhibits a strong AT bias of 91.1%, which is higher than in other Coccoidea insects (82.5% for *D.koreanus* and 84.7% for *S.coffeae*) (Table 2) (Lu et al. 2020, Xu et al. 2021). The PCGs have the lowest AT content (90.3%) and the control region has the highest (92.9%), which differs from all previously-sequenced scale insects (Table 2) (Lu et al. 2020, Xu et al. 2021). The new mitogenome exhibits negative GC-skews (-0.267) and positive AT-skews (0.056), which is common for scale insects (Table 2) and the Hemiptera in general (Wang et al. 2015, Chen et al. 2019).

### Nucleotide diversity and selection pressures

**Figure 4.**
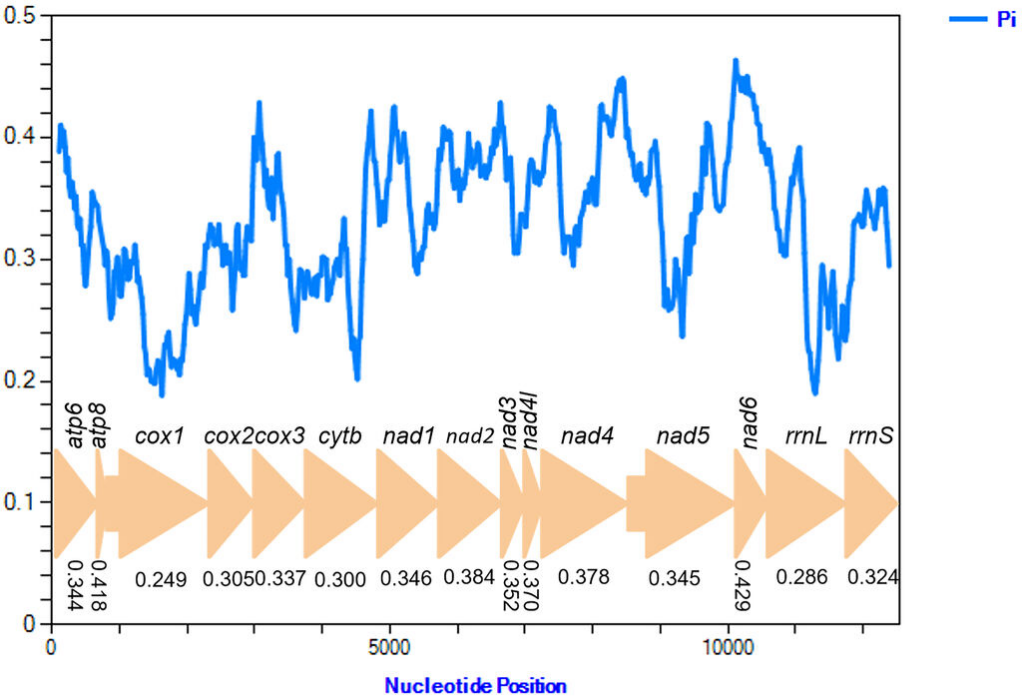
Sliding window analysis of 13 PCGs and two rRNAs of three Coccoidea species. The blue line shows the value of nucleotide diversity Pi (window size = 200 bp, step size = 20 bp). The Pi value for each gene is shown in the graph.

Nucleotide diversity (Pi value) of 13 PCGs and two rRNAs amongst the three scale insect species is shown in Fig. 4. Nucleotide diversity values, calculated for individual genes, ranged from 0.249 (*cox1*) to 0.429 (*nad6*). The results indicated that *atp8* (Pi = 0.418) and *nad6* (Pi = 0.429) had comparatively high nucleotide diversity; *atp6*, *cox2*, *cox3*, *cytb*, *nad1*, *nad2*, *nad3*, *nad4l*, *nad4*, *nad5* and *rrnS* (Pi = 0.300-0.384) exhibited an intermediate nucleotide diversity; and *cox1* (Pi = 0.249) and *rrnL* (Pi = 0.286) were highly conserved genes, with low nucleotide diversity (Fig. 4). The lowest nucleotide diversity in *cox1* is in agreement with observations in most sequenced insect mitogenomes (Ma et al. 2019, Ge et al. 2021, Li et al. 2021, Zhang et al. 2022), which presumably accounts for its ability to provide reliable species identification.

**Figure 5.**
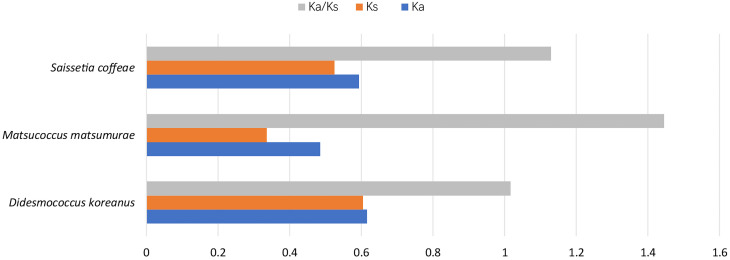
Evolutionary rates of mitochondrial genomes in the three scale insects. The rate of non-synonymous substitutions (Ka), synonymous substitutions (Ks) and the ratio of Ka/Ks were calculated for the PCGs of each mitogenome, using *Aphiscraccivora* as the reference sequence.

The non-synonymous/synonymous (Ka/Ks) substitution ratio can be used to estimate whether a sequence is undergoing purifying, neutral or positive selection. The rates of non-synonymous (Ka) and synonymous substitutions (Ks) and their ratio (Ka/Ks) were calculated for the 13 PCGs of each of the three scale insect species using *Aphiscraccivora* as the reference sequence (Fig. 5). A value of Ka/Ks greater than 1 implies that a gene is evolving predominantly under positive selection. This indicates that non-synonymous mutations are favoured by the Darwinian selection, i.e. that they are retained at a rate greater than synonymous mutations. All Ka/Ks values were above 1, which strongly suggests the presence of positive selection in these species.

### Protein-coding genes

**Figure 6.**
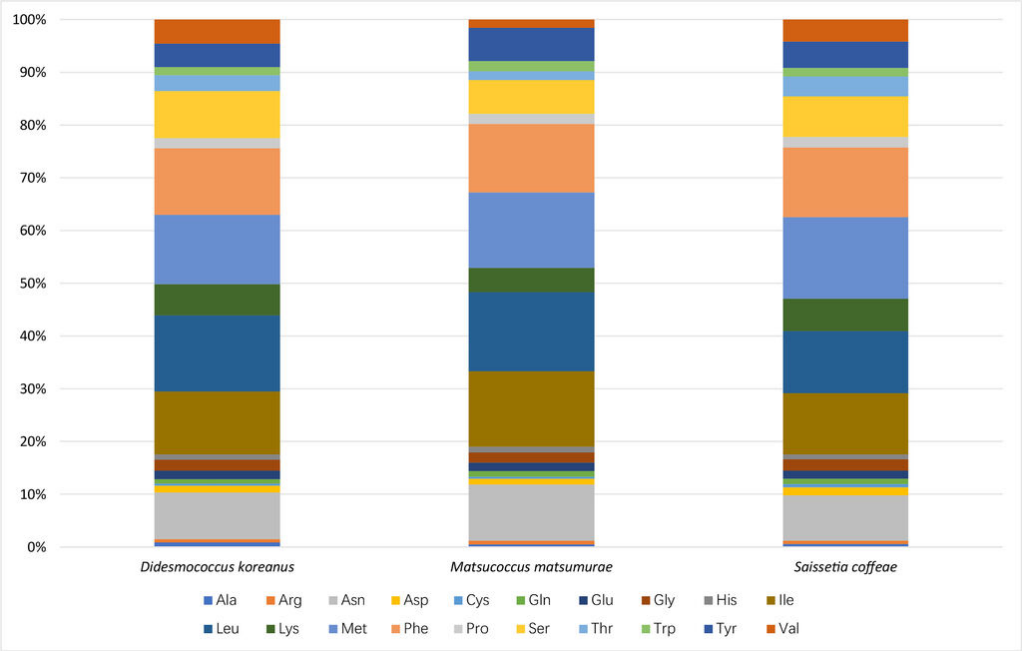
Percentages of each amino acid within the three scale insects. The stop codon is not included.

**Figure 7.**
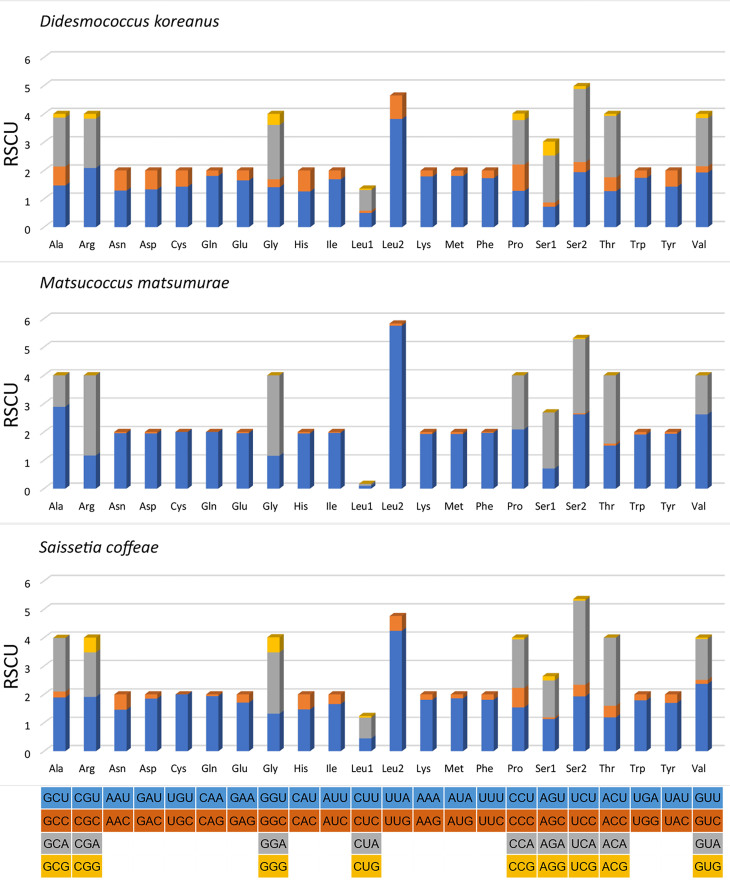
Relative synonymous codon usage (RSCU) in mitogenomes of the three scale insects. The stop codon is not shown.

The 13 PCGs (length: 10,626 bp) account for 69.2% of the complete mitogenome of *M.matsumurae*. All PCGs were initiated by the typical start codon ATN (ATA/T/G/C) and ended with the TAA/G stop codon or their incomplete form T-. This is almost identical to *D.koreanus* (Xu et al. 2021) and *S.coffeae* (Lu et al. 2020). Such incomplete stop codons are common in insects and believed to be completed by post-transcriptional polyadenylation (Ojala et al. 1981). The AT-skews of PCGs were similar (-0.115 to -0.072) amongst the three available scale insects (Table 2). In *M.matsumurae*, the 13 PCGs encode a total of 3,531 amino acids, amongst which the most frequently used are Leu, Met, Ile and Phe (accounting for 56.65% of the total amount), whereas Cys is the least frequently used (0.34%) (Fig. 6). These amino acid usage patterns are very consistent in Coccoidea species (Lu et al. 2020, Xu et al. 2021). Relative synonymous codon usage (RSCU) is summarised in Fig. 7. Results indicate that the four most frequently used codons are UUA (Leu), AUU (Ile), UUU (Phe) and AUA (Met). All of them are composed solely of A or U, which is reflected in the high A+T content of PCGs. Excluding TAA and TAG, 61 codons were observed in *D.koreanus* (no CGC), 46 in *M.matsumurae* (no CUG, GUC, GUG, CCC, CCG, ACG, GCC, GCG, CAG, UGC, CGC, CGG, AGC, AGG, GGC and GGG), and 56 in *S.coffeae* (no CUC, ACG, GCG, UGC, CGC and GGC) (Lu et al. 2020, Xu et al. 2021).

### Transfer RNA genes

**Figure 8.**
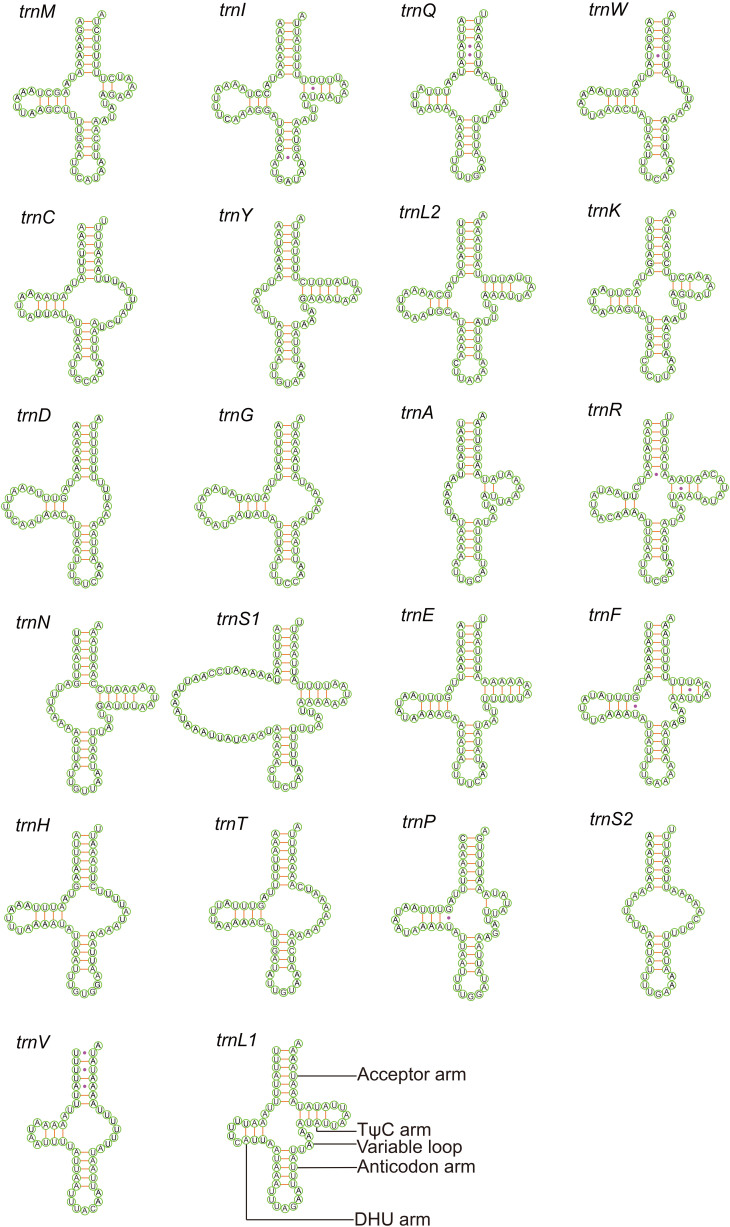
Secondary structures of 22 tRNAs in the mitogenome of *Matsucoccusmatsumurae*. Lines (-) indicate Watson-Crick base pairings, whereas dots (·) indicate unmatched base pairings.

All three sequenced scale insect mitogenomes encode 22 tRNA genes (Suppl. material 3) (Lu et al. 2020, Xu et al. 2021). The AT content of tRNA genes is slightly higher than that of the PCGs, ranging from 87.9% (*D.koreanus*) to 92.3% (*M.matsumurae*) (Table 2). Furthermore, the AT-skew values of tRNAs were all greater than zero (0.043 to 0.054). The length of the 22 tRNA genes ranged from 46 bp (*trnS1* of *D.koreanus*) to 78 bp (*trnS1* of *M.matsumurae*) (Suppl. material 3) (Lu et al. 2020, Xu et al. 2021). Most tRNAs lack either the DHU or TψC arm and some even lost both DHU and TψC arms (Fig. 8) (Lu et al. 2020, Xu et al. 2021). Only ten tRNAs of *D.koreanus*, nine of *M.matsumurae* and 11 of *S.coffeae* could be folded into the common clover-leaf secondary structures (Fig. 8) (Lu et al. 2020, Xu et al. 2021). This suggests that the reduction of DHU or TψC arms of tRNA genes could be a very common phenomenon in the mitogenomes of scale insects. The anticodons of tRNA genes are identical amongst the three scale insects (Fig. 8) (Lu et al. 2020, Xu et al. 2021), except for the *trnE* of *D.koreanus* and *trnK* of *S.coffeae*, which employ UCG and UUU instead of UUC and CUU respectively.

### Ribosomal RNA genes

The AT nucleotide content of *rrnS* and *rrnL* genes of the three scale insects ranges from 86.2% to 92.7% (Table 2). Two rRNAs in these three mitogenomes show a negative AT-skew (-0.099 to -0.027) (Table 2). The *rrnL* gene, located between *trnL1* and *trnV*, ranged in length from 1,161 bp to 1,177 bp and *rrnS* gene, located between *trnV* and *trnI* (*M.matsumurae*) or *trnM* (*D.koreanus* and *S.coffeae*), ranged from 713 bp to 804 bp in length (Fig. 3, Suppl. material 3) (Lu et al. 2020, Xu et al. 2021). Therefore, the length and AT content of rRNAs are conserved in the Coccoidea.

### AT-rich region

**Figure 9.**
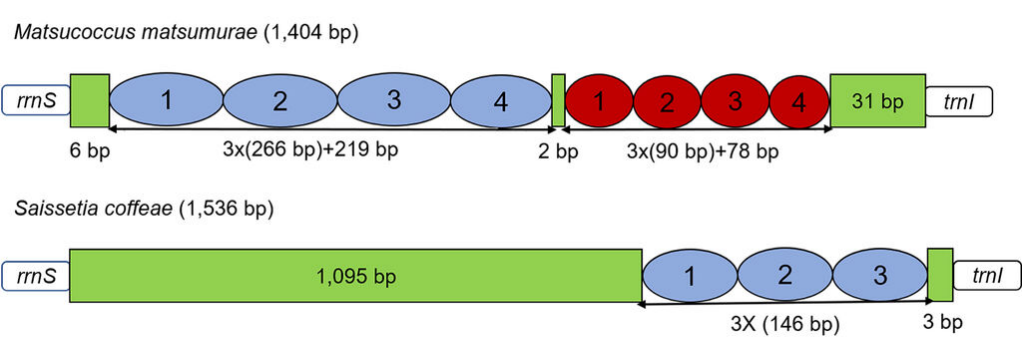
Structures of AT-rich regions in mitogenomes of the three scale insects. The location and copy number of absolute tandem repeat units are represented by blue and red ovals. Green boxes indicate non-repeat regions.

The AT-rich region is believed to be involved in regulating the transcription and replication of DNA in insects (Clayton 1982, Cameron 2014). The AT-rich region of *M.matsumurae* is located between *rrnS* and *trnI*, whereas the AT-rich regions of *D.koreanus* and *S.coffeae* are located between *rrnS* and *trnM* and the size of three scale insects ranges from 1,271 bp to 2,261 bp (Fig. 3, Suppl. material 3) (Lu et al. 2020, Xu et al. 2021). Analyses of the AT-rich regions by the Tandem Repeats Finder indicated that *M.matsumurae* and *S.coffeae* have different numbers of absolute tandem repeat units. Two types of absolute tandem repeats were present in *M.matsumurae* (nucleotide positions: 7 to 1,023 and 1,026 to 1,373 of the AT-rich region). The AT-rich region of *S.coffeae* had only one kind of absolute tandem repeat, located between the nucleotide positions 1,096 to 1,533 (Fig. 9). Like in most insect mitogenomes, tandem repeats are common and the size of tandem repeat regions varies depending on the number of copies of the repeating units (Garey and Wolstenholme 1989). Tandem repeats are thought to play an important role in the control of DNA methylation, gene transcription and replication (Zhang et al. 1995, Huang et al. 2013).

### Phylogenetic relationships

**Figure 10.**
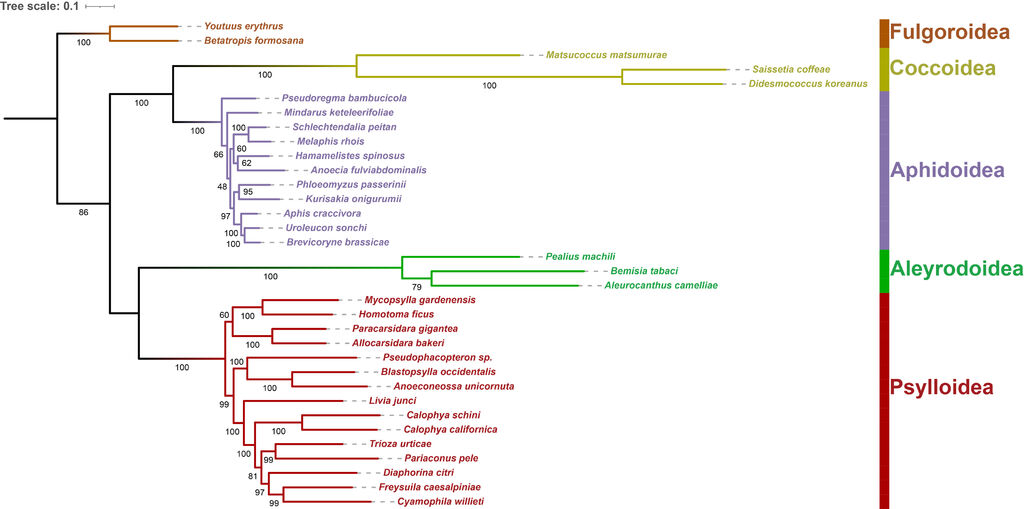
ML phylogenetic tree for Sternorrhyncha, based on the nucleotide sequence data of 37 mitochondrial genes from *Matsucoccusmatsumurae* and other 33 species belonging to five superfamilies of Hemiptera. Bootstrap support values (BS) are indicated on the branches.

**Figure 11.**
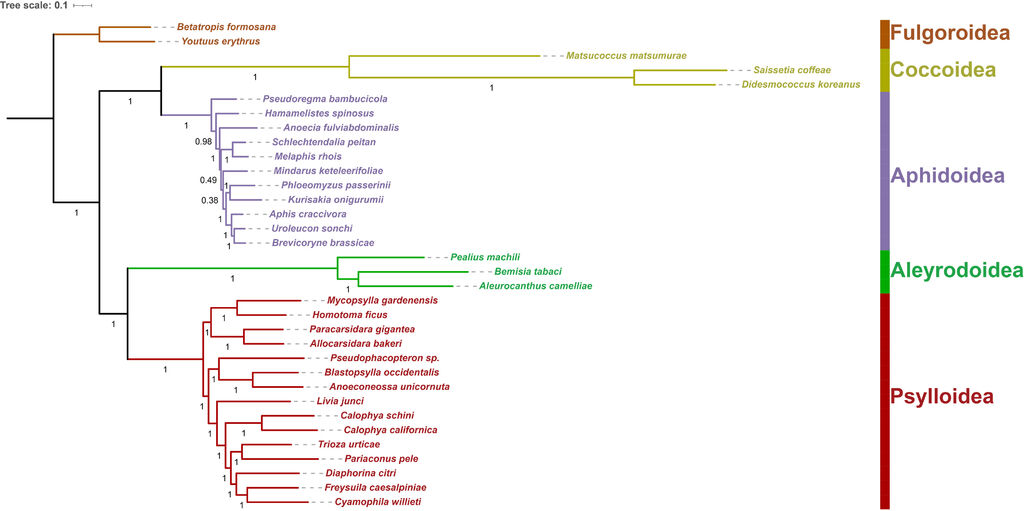
BI phylogenetic tree for Sternorrhyncha, based on the nucleotide sequence data of 37 mitochondrial genes from *Matsucoccusmatsumurae* and other 33 species belonging to five superfamilies of Hemiptera. Bayesian posterior probabilities (PP) are indicated on the branches.

Phylogenetic analyses of 32 species of Sternorrhyncha, including two outgroups, based on ML and BI analyses of nucleotide sequence data of 37 mitochondrial genes, yielded largely congruent topologies, with most branches receiving strong support (Figs 10, 11). As proposed by previous studies (Lu et al. 2020, Xu et al. 2021), the relationships amongst sternorrhynchan superfamilies are inferred as ((Coccoidea + Aphidoidea) + (Aleyrodoidea + Psylloidea)).

All analyses consistently recovered the monophyly of Sternorrhyncha and its four superfamilies (Coccoidea, Aphidoidea, Aleyrodoidea and Psylloidea) with strong support (BS = 86; PP = 1.00) (Figs 10, 11Suppl. material 4). Furthermore, *M.matsumurae* clustered with the other two coccoid species, *D.koreanus* and *S.coffeae*, with strong support (BS = 100; PP = 1.00) (Figs 10, 11Suppl. material 4), thus confirming its affiliation with the superfamily Coccoidea. Within the clade of Coccoidea, *M.matsumurae* is positioned as the earliest branching taxon in both nucleotide and amino acid trees, which suggests that Matsucoccidae is a more ancient taxon than Coccidae.

## Funding

This study was supported by the Projects of Guizhou Science and Technology Platform and Talent Team (QKHPTRC [2016] 5669) and Survey and Research on Exotic Species in Guizhou Province (AS [2017] 09, KJZXSA [2018] 016, and KJZXSA [2019] 014).

## Acknowledgements

We thank Ivan Jakovlić (Bio-Transduction Lab, Wuhan, China) for proofreading of the manuscript and giving us valuable advice.

## Supplementary Material

20BE5020-23F3-5D84-908C-B44DE1096D47 10.3897/BDJ.10.e94915.suppl1 Supplementary material 1 Partitioning schemes and substitution models used for ML phylogenetic analyses
Data type Table Brief description The best partitioning schemes and substitution models for PCG123 + tRNA + rRNA dataset comprising 13 PCGs, 22 tRNAs and two rRNAs of 34 species of Hemiptera used for ML phylogenetic analyses File: oo_759534.docx https://binary.pensoft.net/file/759534 Kai Hu and Jiansheng Qiu

4233D680-E920-5F45-9270-B6F573DEBDBD 10.3897/BDJ.10.e94915.suppl2 Supplementary material 2 Partitioning schemes and substitution models used for BI phylogenetic analyses
Data type Table Brief description The best partitioning schemes and substitution models for PCG123 + tRNA + rRNA dataset comprising 13 PCGs, 22 tRNAs and two rRNAs of 34 species of Hemiptera used for BI phylogenetic analyses File: oo_759535.docx https://binary.pensoft.net/file/759535 Kai Hu and Jiansheng Qiu

1A58C0DE-B717-5AD4-8D3E-E614A360A2A3 10.3897/BDJ.10.e94915.suppl3 Supplementary material 3 Mitogenomic organisation of *Matsucoccusmatsumurae* Data type Table File: oo_742094.docx https://binary.pensoft.net/file/742094 Kai Hu and Jiansheng Qiu

856E1681-E719-5468-866F-29A73C9A1C3A 10.3897/BDJ.10.e94915.suppl4 Supplementary material 4 BI phylogenetic tree for Sternorrhyncha Data type Figure Brief description BI phylogenetic tree for Sternorrhyncha, based on the amino acid sequence data of 13 PCGs from Matsucoccusmatsumurae and other 33 species belonging to five superfamilies of Hemiptera. Bayesian posterior probabilities (PP) are indicated on the branches File: oo_759530.jpg https://binary.pensoft.net/file/759530 Kai Hu and Jiansheng Qiu

## Figures and Tables

**Table 1.** Mitogenomes of the 34 Hemiptera insects used in this study.

Superfamily	Family	Species	Accession number	Reference
Aleyrodoidea	Aleyrodidae	*Pealiusmachili*	MT015588	Zhang et al. (2020)
		*Bemisiatabaci*	MK386668	Kunz et al. (2019)
		*Aleurocanthuscamelliae*	KU761949	Chen et al. (2016)
Psylloidea	Calophyidae	*Calophyaschini*	MF431591	Percy et al. (2018)
		*Calophyacalifornica*	NC_036302	Unpublished
	Carsidaridae	*Paracarsidaragigantea*	NC_038112	Percy et al. (2018)
		*Allocarsidarabakeri*	NC_038107	Percy et al. (2018)
	Homotomidae	*Mycopsyllagardenensis*	MF443235	Percy et al. (2018)
		*Homotomaficus*	MG989227	Percy et al. (2018)
	Liviidae	*Diaphorinacitri*	MF614824	Unpublished
		*Liviajunci*	MG989230	Percy et al. (2018)
	Phacopteronidae	*Pseudophacopteron* sp.	MG989234	Percy et al. (2018)
	Psyllidae	*Cyamophilawillieti*	MN364946	Song et al. (2019)
		*Freysuilacaesalpiniae*	NC_038135	Percy et al. (2018)
	Triozidae	*Triozaurticae*	MG989240	Percy et al. (2018)
		*Pariaconuspele*	MG989233	Percy et al. (2018)
	Aphalaridae	*Anoeconeossaunicornuta*	NC_038108	Percy et al. (2018)
		*Blastopsyllaoccidentalis*	NC_038147	Percy et al. (2018)
Aphidoidea	Anoeciidae	*Anoeciafulviabdominalis*	KP722588	Unpublished
	Aphididae	*Uroleuconsonchi*	MT533446	Unpublished
		*Aphiscraccivora*	MT095075	Voronova et al. (2020)
		*Brevicorynebrassicae*	MT900510	An et al. (2021)
	Hormaphididae	*Hamamelistesspinosus*	NC_050942	Lu et al. (2020)
		*Pseudoregmabambucicola*	MK847518	Zhang et al. (2019)
	Mindaridae	*Mindarusketeleerifoliae*	NC_033410	Unpublished
	Pemphigidae	*Schlechtendaliapeitan*	NC_059063	Unpublished
		*Melaphisrhois*	KY624581	Ren and Wen (2017)
	Phloeomyzidae	*Phloeomyzuspasserinii*	KP722571	Unpublished
	Thelaxidae	*Kurisakiaonigurumii*	KP722578	Unpublished
Coccoidea	Coccidae	*Didesmococcuskoreanus*	NC_057479	Xu et al. (2021)
		*Saissetiacoffeae*	MN863803	Lu et al. (2020)
	Matsucoccidae	*Matsucoccusmatsumurae*	OM396907	This study
Fulgoroidea	Achilidae	*Betatropisformosana*	MH324927	Unpublished
	Caliscelidae	*Youtuuserythrus*	NC_059811	Gong et al. (2021)

**Table 2.** Nucleotide composition and skewness of mitogenomes of *Didesmococcuskoreanus*, *Matsucoccusmatsumurae* and *Saissetiacoffeae*.

Feature	Length	A+T%	AT-skew	GC-skew
*D.koreanus* / *M.matsumurae* / *S.coffeae*				
Whole genome	15143/15360/15389	82.5/91.1/84.7	0.142/0.056/0.189	-0.357/-0.267/-0.369
PCGs	10599/10626/10632	81.9/90.3/84.1	-0.084/-0.115/-0.072	-0.108/0.016/-0.107
tRNAs	1300/1330/1373	87.9/92.3/89.3	0.043/0.054/0.054	0.019/-0.059/-0.007
rRNAs	1914/1890/1965	86.2/92.7/87.2	-0.027/-0.062/-0.099	0.426/0.304/0.440
Control region	1350/1404/1536	77.7/92.9/82.1	-0.071/0.015/-0.045	-0.252/-0.818/-0.295
