# Supplementary material for: The first complete mitochondrial genome of Matsucoccidae (Hemiptera, Coccoidea) and implications for its phylogenetic position
Source: Biodivers Data J. 2022 Nov 9;10:e94915. doi: 10.3897/BDJ.10.e94915 (PMC9836553; doi:10.3897/BDJ.10.e94915)
Supplement: Supplementary material 1 — Partitioning schemes and substitution models used for ML phylogenetic analyses [file bdj-10-e94915-s001.docx]

**Table S1.** The best partitioning schemes and substitution models for PCG123 + tRNA + rRNA dataset comprising 13 PCGs, 22 tRNAs and two rRNAs of 34 species of Hemiptera used for ML phylogenetic analyses.

| Optimal partition | Model | Subset partition |
| --- | --- | --- |
| Partition1 | GTR + I + G | cox3, *atp6*, *nad3* |
| Partition2 | GTR + I + G | *atp8*, *nad2*, *nad6* |
| Partition3 | TIM + I + G | *cox1* |
| Partition4 | GTR + I + G | *cox2*, *cytb* |
| Partition5 | GTR + I + G | *nad1*, *nad4L*, *nad4*, *nad5* |
| Partition6 | GTR + I + G | *rrnL*, *trnL1*, *rrnS* |
| Partition7 | HKY + G | *trnV*, *trnQ*, *trnA*, *trnC* |
| Partition8 | GTR + G | *trnW*, *trnI*, *trnM*, *trnR*, *trnT*, *trnS2*, *trnK*, *trnN*, *trnS1*, *trnE*, *trnG*, *trnY*, *trnP*, *trnF*, *trnL2*, *trnD*, *trnH* |
