# Supplementary material for: The first complete mitochondrial genome of Matsucoccidae (Hemiptera, Coccoidea) and implications for its phylogenetic position
Source: Biodivers Data J. 2022 Nov 9;10:e94915. doi: 10.3897/BDJ.10.e94915 (PMC9836553; doi:10.3897/BDJ.10.e94915)
Supplement: Supplementary material 2 — Partitioning schemes and substitution models used for BI phylogenetic analyses [file bdj-10-e94915-s002.docx]

**Table S2.** The best partitioning schemes and substitution models for PCG123 + tRNA + rRNA dataset comprising 13 PCGs, 22 tRNAs and two rRNAs of 34 species of Hemiptera used for BI phylogenetic analyses.

| Optimal partition | Model | Subset partition |
| --- | --- | --- |
| Partition1 | GTR + I + G | cox3, *atp6*, *nad3* |
| Partition2 | GTR + I + G | *atp8*, *nad6*, *nad2* |
| Partition3 | GTR + I + G | *cox1* |
| Partition4 | GTR + I + G | *cytb*, *cox2* |
| Partition5 | GTR + I + G | *nad1*, *nad4L*, *nad4*, *nad5* |
| Partition6 | GTR + I + G | *trnL1*, *rrnL*, *rrnS* |
| Partition7 | HKY+G | *trnQ, trnV, trnA, trnC* |
| Partition8 | GTR+G | *trnS2, trnS1, trnT, trnK, trnM, trnI, trnN, trnL2, trnR, trnD, trnW, trnP* |
| Partition9 | HKY+G | *trnH, trnY, trnF, trnE, trnG* |
