# Supplementary material for: The first complete mitochondrial genome of Matsucoccidae (Hemiptera, Coccoidea) and implications for its phylogenetic position
Source: Biodivers Data J. 2022 Nov 9;10:e94915. doi: 10.3897/BDJ.10.e94915 (PMC9836553; doi:10.3897/BDJ.10.e94915)
Supplement: Supplementary material 3 — Mitogenomic organisation of Matsucoccusmatsumurae [file bdj-10-e94915-s003.docx]

**Table S3.** Mitogenomic organization of *Matsucoccus matsumurae*.

| Gene name | Location | | Size (bp) | Intergenic  nucleotides | Codon | | Strand |
| --- | --- | --- | --- | --- | --- | --- | --- |
|  | From | To |  |  | Start | Stop |  |
| *trnI* | 1 | 67 | 67 |  |  |  | H |
| *trnQ* | 72 | 124 | 53 | 4 |  |  | L |
| *trnM* | 127 | 188 | 62 | 2 |  |  | H |
| *nad2* | 189 | 1127 | 939 |  | ATT | TAA | H |
| *trnW* | 1133 | 1189 | 57 | 5 |  |  | H |
| *trnY* | 1210 | 1267 | 58 | 20 |  |  | L |
| *trnC* | 1273 | 1330 | 58 | 5 |  |  | L |
| *cox1* | 1333 | 2856 | 1524 | 2 | ATT | TAA | H |
| *trnL2* | 2865 | 2930 | 66 | 8 |  |  | H |
| *cox2* | 2931 | 3594 | 664 |  | ATA | T | H |
| *trnK* | 3595 | 3657 | 63 |  |  |  | H |
| *trnD* | 3657 | 3714 | 58 | -1 |  |  | H |
| *atp8* | 3719 | 3826 | 108 | 4 | ATA | TAA | H |
| *atp6* | 3823 | 4449 | 627 | -4 | ATA | TAA | H |
| *cox3* | 4450 | 5202 | 753 |  | ATG | TAA | H |
| *trnG* | 5253 | 5313 | 61 | 50 |  |  | H |
| *nad3* | 5314 | 5664 | 351 |  | ATA | TAA | H |
| *trnA* | 5665 | 5717 | 53 |  |  |  | H |
| *trnR* | 5705 | 5771 | 67 | -13 |  |  | H |
| *trnN* | 5799 | 5857 | 59 | 27 |  |  | H |
| *trnS1* | 5836 | 5913 | 78 | -22 |  |  | H |
| *trnE* | 5906 | 5970 | 65 | -8 |  |  | H |
| *trnF* | 5953 | 6016 | 64 | -18 |  |  | L |
| *nad5* | 6035 | 7669 | 1635 | 18 | ATT | TAA | L |
| *trnH* | 7670 | 7728 | 59 |  |  |  | L |
| *nad4* | 7752 | 9035 | 1284 | 23 | ATG | TAA | L |
| *nad4L* | 9029 | 9310 | 282 | -7 | ATT | TAA | L |
| *trnT* | 9312 | 9370 | 59 | 1 |  |  | H |
| *trnP* | 9361 | 9421 | 61 | -10 |  |  | L |
| *nad6* | 9423 | 9849 | 427 | 1 | ATT | T | H |
| *cytb* | 9848 | 10981 | 1134 | -2 | ATT | TAA | H |
| *trnS2* | 10986 | 11033 | 48 | 4 |  |  | H |
| *nad1* | 11053 | 11952 | 900 | 19 | ATT | TAA | L |
| *trnL1* | 11953 | 12014 | 62 |  |  |  | L |
| *rrnL* | 12015 | 13191 | 1177 |  |  |  | L |
| *trnV* | 13192 | 13243 | 52 |  |  |  | L |
| *rrnS* | 13244 | 13956 | 713 |  |  |  | L |
| Control region | 13957 | 15360 | 1404 |  |  |  | H |
