# Supplementary figures and images for: The first complete mitochondrial genome of Matsucoccidae (Hemiptera, Coccoidea) and implications for its phylogenetic position
Source: Biodivers Data J. 2022 Nov 9;10:e94915. doi: 10.3897/BDJ.10.e94915 (PMC9836553; doi:10.3897/BDJ.10.e94915)

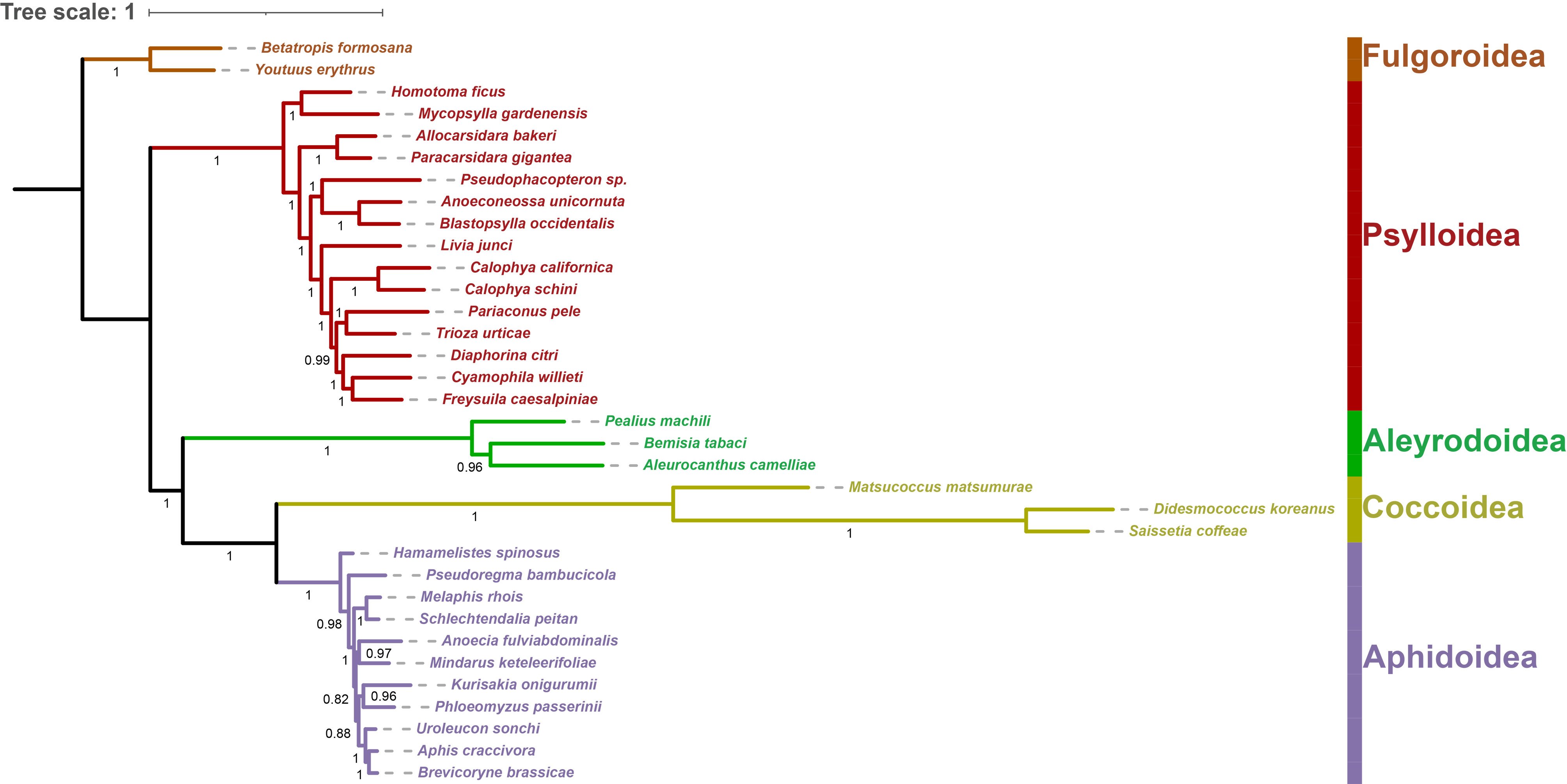

Supplement: Supplementary material 4 — BI phylogenetic tree for Sternorrhyncha [file bdj-10-e94915-s004.jpg]
